# Non-Alcoholic Fatty Liver Disease (NAFLD) and Potential Links to Depression, Anxiety, and Chronic Stress

**DOI:** 10.3390/biomedicines9111697

**Published:** 2021-11-16

**Authors:** Sue Shea, Christos Lionis, Chris Kite, Lou Atkinson, Surinderjeet S. Chaggar, Harpal S. Randeva, Ioannis Kyrou

**Affiliations:** 1 Warwick Medical School, University of Warwick, Coventry CV4 7AL, UK; sue.shea@warwick.ac.uk; 2 Warwickshire Institute for the Study of Diabetes, Endocrinology and Metabolism (WISDEM), University Hospitals Coventry and Warwickshire NHS Trust, Coventry CV2 2DX, UK; c.kite@chester.ac.uk (C.K.); l.atkinson1@aston.ac.uk (L.A.); 3 Clinic of Social and Family Medicine, School of Medicine, University of Crete, 71003 Heraklion, Greece; lionis@galinos.med.uoc.gr; 4 Centre for Active Living, University Centre Shrewsbury, University of Chester, Shrewsbury SY3 8HQ, UK; 5 Centre for Sport, Exercise and Life Sciences, Research Institute for Health & Wellbeing, Coventry University, Coventry CV1 5FB, UK; 6 School of Psychology, College of Health and Life Sciences, Aston University, Birmingham B4 7ET, UK; 7 Sowe Valley Primary Care Network, Forum Health Centre, Coventry CV2 5EP, UK; surinder.chaggar@nhs.net; 8 Aston Medical School, College of Health and Life Sciences, Aston University, Birmingham B4 7ET, UK; 9 Department of Food Science and Human Nutrition, Agricultural University of Athens, 11855 Athens, Greece

**Keywords:** non-alcoholic fatty liver disease, NAFLD, NASH, metabolic syndrome, insulin resistance, obesity, depression, anxiety, stress, health related quality of life

## Abstract

Non-alcoholic fatty liver disease (NAFLD) constitutes the most common liver disease worldwide, and is frequently linked to the metabolic syndrome. The latter represents a clustering of related cardio-metabolic components, which are often observed in patients with NAFLD and increase the risk of cardiovascular disease. Furthermore, growing evidence suggests a positive association between metabolic syndrome and certain mental health problems (e.g., depression, anxiety, and chronic stress). Given the strong overlap between metabolic syndrome and NAFLD, and the common underlying mechanisms that link the two conditions, it is probable that potentially bidirectional associations are also present between NAFLD and mental health comorbidity. The identification of such links is worthy of further investigation, as this can inform more targeted interventions for patients with NAFLD. Therefore, the present review discusses published evidence in relation to associations of depression, anxiety, stress, and impaired health-related quality of life with NAFLD and metabolic syndrome. Attention is also drawn to the complex nature of affective disorders and potential overlapping symptoms between such conditions and NAFLD, while a focus is also placed on the postulated mechanisms mediating associations between mental health and both NAFLD and metabolic syndrome. Relevant gaps/weaknesses of the available literature are also highlighted, together with future research directions that need to be further explored.

## 1. Introduction

Non-alcoholic fatty liver disease (NAFLD) currently constitutes the most common chronic liver disease worldwide, representing a “silent epidemic” [1,2]. Indeed, NAFLD prevalence rates of up to 25–30% have been reported in the general population (depending on the studied population/cohort and the applied diagnostic criteria/method), with increasing trends in both developed and developing countries [2,3,4]. Of note, markedly higher NAFLD prevalence is noted among adults with obesity [2,3,4], and is estimated to affect around 70–90% of this population [5]. Given this strong link to obesity, NAFLD is often referred to as the hepatic manifestation of metabolic syndrome [6,7,8,9]. Denoting a clustering of interrelated conditions revolving around obesity-related insulin resistance, metabolic syndrome may include central/abdominal obesity, type 2 diabetes (T2DM), hypertension, and dyslipidemia [10]. Overall, the escalating prevalence of obesity in recent decades has led to a dramatic increase in the global prevalence of both metabolic syndrome and NAFLD, with almost 85% of NAFLD patients also presenting with various metabolic syndrome components (e.g., insulin resistance and obesity) [3,6,11,12]. Moreover, metabolic syndrome and its associated cardio-metabolic diseases, including NAFLD, are independently related to a substantially increased risk of developing cardiovascular disease (CVD) [10,13,14].

Compelling evidence from clinical and epidemiological studies indicates that the components of metabolic syndrome share common underlying pathogenetic mechanisms, which are mostly—but not exclusively—driven by obesity and obesity-related insulin resistance [10,12,15,16]. Because of the strong associations between metabolic syndrome and NAFLD, the full spectrum of risk factors and the underlying mechanisms that link them are currently under extensive research [3,10,12,17]. As such, it should be highlighted that growing evidence supports a direct association between metabolic syndrome and common mental health disorders (e.g., depression and anxiety) [18,19,20], in addition to its well-established associations with other cardio-metabolic conditions. Furthermore, these affective disorders, as well as chronic stress, have been linked to key components of metabolic syndrome, such as obesity and T2DM [21,22,23], which may further adversely impact cardio-metabolic health and health-related quality of life (HRQoL). Indeed, studies in both humans and animal models provide growing evidence of the risk of metabolic syndrome in cases where depressive disorders are present [19,23].

Despite the above noted associations, and although high prevalence rates of such mood disorders (e.g., depression) have been identified among patients with NAFLD [24,25], less work has focused on the potential relationship between NAFLD and mental health. Given the aforementioned close links between NAFLD and metabolic syndrome, the potential bidirectional associations between NAFLD and common mental health disorders that may coexist in patients with NAFLD merit further investigation. In this context, it should also be highlighted that the concept of comorbidity can be difficult to define. Accordingly, a relevant review by Valderas et al. [26] draws attention to the fact that the concept of comorbidity might overlap with other related constructs, such as the burden of disease and multi-morbidity, further suggesting that there are a number of distinctions pertaining to this concept. For example, it is important to consider the nature of the condition and the basis for classification, as certain conditions may form part of a spectrum rather than existing as separate entities. The importance of the condition also represents a key factor in terms of ascertaining which disorder might represent the core or ‘index’ disorder, and which might be referred to as the ‘comorbid’ condition when two or more conditions co-exist.

Taking into account the above, the present review examined published research that indicates the presence of associations between NAFLD and highly prevalent mental health disorders, namely depression, anxiety, and chronic stress, as well as impaired HRQoL, which is closely linked to mental health wellbeing. The aim of this review is to provide a comprehensive overview of the current evidence on the potential relationship(s) between these common mental health problems and both NAFLD and metabolic syndrome, keeping in mind the complexity of the concept of comorbidity. Thus, this review will begin by discussing current evidence in relation to depression, anxiety, and chronic psychosocial stress within the context of associations with metabolic syndrome and NAFLD. Then, associations between impaired HRQoL and both the metabolic syndrome and NAFLD are also summarized. Finally, the concluding sections of this review also focus on key underlying mechanisms that are considered to mediate associations between NAFLD, metabolic syndrome, and mental health, as well as on the gaps/weaknesses of the relevant published literature, and the future research directions which need to be further explored. The main studies included in this review are summarized in Tables 1 and 2.

## 2. Methods

Although this work presents a narrative review, rather than a systematic review, a predefined search strategy was formulated and was applied to identify relevant papers in the English language, utilizing both relevant search terms and medical subject headings (MeSH) [27], which related to NAFLD, metabolic syndrome, depression, anxiety, stress, and HRQoL. The searched databases included both PubMed and Google Scholar, focusing predominantly on work published over the past decade. After removing duplicates and screening, key papers that reported evidence on identified associations between NAFLD or metabolic syndrome and depression, anxiety, stress, and HRQoL were reviewed in full and were included as relevant to the scope of this review, as presented in the following sections.

## 3. Depression

Often accompanied by rumination and cognitive impairment, depression is a highly prevalent illness, which affects more than a quarter of a billion people of all ages and constitutes a leading cause of years lived with disability globally [14,28,29].

### 3.1. Depression/Anxiety and Metabolic Syndrome

It is now recognized that depression and/or anxiety are associated with increased risk of CVD and metabolic syndrome components (e.g., depression exhibits an independent association to 10-year CVD incidence, and increases the risk of T2DM by up to 60%) [21,30,31]. Links between depression/anxiety and increased likelihood of alcohol overconsumption and poor dietary and/or exercise habits are considered among the possible mediators of these associations [32]. This is further supported by systematic review data showing that affective disorders, such as depression, can double the risk of metabolic syndrome, partially owing to the poor health-related behaviors associated with depression [14]. Although significant associations between depression and metabolic syndrome are supported by such data, a potential causal bidirectional relationship remains to be fully elucidated, as depression may promote metabolic syndrome, while factors related to metabolic syndrome—either psychological (e.g., obesity-related stigma) or biological (e.g., increased activation of pro-inflammatory pathways representative of the stress response system)—may also lead to depression [14,23].

Furthermore, various studies have demonstrated positive associations between metabolic syndrome and anxiety, indicating that, in addition to depression, anxiety may also be significantly more prevalent in individuals with metabolic syndrome compared with those without [33,34]. This significant association of anxiety with metabolic syndrome is also supported by systematic review and meta-analysis data, which pooled findings from 18 relevant cross-sectional studies [35]. However, other comorbidities and their related symptoms may overlap with symptoms included in tools designed to measure depression and anxiety. Indeed, some earlier studies have been unable to detect significant associations between metabolic syndrome and anxiety [36,37]. Likewise, in the two cohort studies included in the systematic review by Tang, Wang, and Lian [35], no significant association was found between anxiety and metabolic syndrome [38,39]. Interestingly, a cross-sectional study by Akbari et al. [40], utilizing a sample from the Isfahan Cohort Study in Iran, reported a negative relationship between anxiety and metabolic syndrome, with a lower prevalence of metabolic syndrome in patients with anxiety compared with those without. This finding contradicts the reported outcomes of the aforementioned studies, and may be potentially attributed to the applied methodology, with the study authors drawing attention to the use of a self-reported tool for measuring anxiety and depression (i.e., the hospital anxiety and depression scale, HADS), rather than the application of more rigid diagnostic methods.

### 3.2. Depression/Anxiety and NAFLD

Given the identified relationships between affective disorders and metabolic syndrome, it is plausible that similar positive associations may exist between such mental health conditions and NAFLD. However, Weinstein et al. [41] report that, despite the recognition that depression is associated with many medical conditions, much less attention has been directed towards patients with chronic liver disease. With this in mind, these authors conducted a study aimed at comparing the prevalence of depression in individuals with NAFLD, hepatitis B, and hepatitis C, utilizing data extracted from a database containing clinical and self-reported depression data for CLD patients. This study identified a higher prevalence of depression in patients with NAFLD and hepatitis C, compared with patients with hepatitis B and members of the general population.

In a study by Youssef et al. [42], the potential association between depression/anxiety and the histological features of NAFLD among 567 patients diagnosed with NAFLD was explored. Subclinical depression was identified in 53% of these patients, while clinical depression was observed in 14%. Similarly, subclinical and clinical anxiety was observed in 45% and 25% of these patients, respectively. Notably, this study identified a positive association between greater hepatocyte ballooning and depression in patients with NAFLD. Although the exact underlying mechanisms for this association remain unclear, it is noteworthy that the clinical and lifestyle variables measured in this study, including body mass index (BMI) and hypertension, were associated with the severity of both depression and anxiety [42]. Another interesting study by Jung et al. [43], involving a Korean population, further sought to investigate the extent to which the risk for depression might be associated with the severity of NAFLD. Indeed, evidence of an association between the severity of NAFLD and depression emerged from this study, leading the authors to suggest that advanced stages of NAFLD potentially have a greater association with depression [43]. Similarly, a recent retrospective cross-sectional study by Choi et al. [44] identified that severe steatosis is significantly related to both state and trait anxiety in patients with NAFLD. However, this study did not observe associations between NAFLD and depression until gender was taken into consideration, whereby a 44% increase in the risk of depression among women with NAFLD was demonstrated.

In a small cohort study conducted in Pakistan, the frequency of depression, as assessed by the Patient Health Questionnaire (PHQ-9), in 170 patients with ultrasound-diagnosed NAFLD was investigated [45]. Almost one in four participants of this study were identified as having depression, with no significant impact of gender, age, or socioeconomic status on depression in these NAFLD patients. Despite the small sample size, this study suggests that depression appears more frequently in individuals with NAFLD, highlighting the need for further research in this field. Indeed, using data from a large sample of 10,484 individuals in the United States, Kim et al. [46] also identified a higher prevalence of depression among individuals with NAFLD compared with those without. This study further revealed that subjects with depression were at a higher risk of developing NAFLD. Although individuals with depression were of older age and had other comorbidities, such as diabetes and hypertension, as well as higher total cholesterol and BMI, the findings of this study indicate that depression may represent an independent risk factor in relation to NAFLD [46].

Moreover, the longitudinal association between depression and the risk of NAFLD was examined by Cho et al. [47] in a cohort of 142,005 Korean adults without excessive alcohol consumption or hepatic steatosis at baseline that were followed for up to 8.9 years [47]. The findings of this study showed that depression at baseline, as assessed by the validated Center for Epidemiologic Studies-Depression score (CES-D), was associated with an increased risk of incident hepatic steatosis diagnosed by ultrasonography, as well as a higher probability of advanced liver fibrosis, particularly among individuals with obesity. In another large study involving a sample of 19,871 patients with NAFLD and a matched control group of 19,871 individuals, Labenz et al. [48] investigated associations between NAFLD and depression and anxiety over the course of a 10-year period. Over this study timeframe, the percentage of individuals diagnosed with depression was 21.2% and 18.2% for those with and without NAFLD, respectively, while the corresponding anxiety rates were 7.9% and 6.5%, respectively. Furthermore, this study identified a significant association between NAFLD and an initial antidepressant prescription [48]. Although this study was unable to prove causality, a significant association between NAFLD and development of depression/anxiety was noted, independent of comorbidities.

A further important point for the clinical practice is that depressive disorders may also have an impact on the treatment outcomes in patients with NAFLD. This was evident in a study by Tomeno et al. [25], which explored the effects of a 48-week lifestyle intervention in patients with NAFLD presenting with or without major depressive disorder (MDD). Of the 258 patients participating in this study, 32 presented with MDD in addition to NAFLD, and these patients exhibited a poor response with less effective treatment outcomes. The poor adherence/outcomes noted in this study could be due to psychological factors related to depression, including effects on memory and self-efficacy [25]. This suggests that multi-component lifestyle modification programmes may be required in patients presenting with both NAFLD and MDD [25].

Overall, depression has been identified as one of the most common extrahepatic diseases associated with NAFLD [49] and, collectively, the aforementioned evidence suggests a potential bidirectional association of depression and anxiety with the development, and even the severity, of NAFLD. However, other underlying factors may be either mediators or confounders in these associations, including the well-established links between depression/anxiety and unhealthy lifestyle behaviors, and the co-existence of other cardio-metabolic comorbidities. Indeed, a recent systematic review and meta-analysis highlights several risk factors (including BMI, diabetes, and being female) associated with a high prevalence of depression in NAFLD patients [50]. Therefore, it remains unclear whether depression and/or anxiety can be considered independent risk factors for the development and progression of NAFLD, and vice versa. Thus, the existing evidence warrants further investigations aiming to clarify the extent and direction(s) of the potential associations between depression/anxiety and NAFLD.

## 4. Chronic Psychosocial Stress

The concept of stress has been linked with physical health conditions for a considerable time, because, as reported by Selye, we have had some notion of the concept of stress ever since the word ‘disease’ was first used [51]. To date, psychosocial stress is known to be associated with increased prevalence rates of a number of cardio-metabolic diseases, including obesity, T2DM, hypertension and CVD [23,52]. Of note, chronic psychosocial stress poses a risk factor for obesity, with evidence suggesting that this might be reflective of exposure to a wide range of different stressors rather than resulting from a single stressor [23,53].

### 4.1. Chronic Psychosocial Stress and Metabolic Syndrome

Chronic psychosocial stress has frequently been reported as a risk factor for metabolic syndrome, partially associated with disturbances in metabolic homeostasis [21,22,23,54,55,56]. Therefore, the cardio-metabolic effects of chronic psychosocial stress in the longer term can be highly damaging [23,31].

In a systematic review, Kuo et al. [57] sought to further establish whether the association between stress and metabolic syndrome differed depending on the source of stress. The meta-analysis of the available data showed that the weakest effect was noted for general stress, while the strongest effect was evident for occupational stress. These authors concluded that research should investigate the impact of frequently experienced different sources of stress on metabolic consequences, and suggested that the usual methods for the prevention of metabolic syndrome, such as lifestyle changes, may be less effective if psychosocial stress is overlooked [57].

### 4.2. Chronic Psychosocial Stress and NAFLD

Limited research studies have explored relationships between stress and NAFLD, although an underlying association is considered likely [56]. Indeed, stress involves both behavioral and biological responses, which activate the hypothalamic–pituitary–adrenal (HPA) axis, resulting in elevated levels of cortisol and pro-inflammatory biomarkers that could be involved in the development of NAFLD [56].

Notably, in exploring correlations between NAFLD, dietary habits, stress, and HRQoL in Korean individuals, Han [58] identified a 1.3-fold increase in the risk of NAFLD in individuals with increased perceived stress, suggesting that stress management should be included in the treatment of NAFLD [58]. Furthermore, a large cross-sectional study involving 171,321 apparently healthy Korean adults identified an independent association between higher levels of perceived stress and a higher prevalence of NAFLD [56]. This association was stronger among men and individuals with obesity in comparison with women and those with normal body weight, while it remained significant after adjusting for multiple metabolic, behavioral and socioeconomic factors. Indeed, although stress is often linked to unhealthy behaviors, the noted association was still significant even after controlling for such risk factors (e.g., smoking, lack of physical activity, and alcohol consumption), highlighting that further research is needed to fully ascertain the potential mediating mechanisms [56].

In addition, meta-analysis data from 16 prospective studies in the U.K. general population with 166,631 individuals and a follow-up period of approximately 9.5 years [9] showed that psychological distress was associated with significantly increased liver disease mortality, together with increased scores on the 12-item version of the General Health Questionnaire (GHQ-12). Given that psychological distress is a risk factor for CVD [59,60], these findings suggest that it could also play a role in the development of liver disease. Furthermore, because the association between liver disease and psychological distress could not be totally explained by lifestyle habits, BMI, socioeconomic status, or the co-existence of diabetes, this study concluded that other underlying mechanisms might be also partially responsible for this link [9].

In view of the evidence suggesting that both occupational stress and NAFLD represent growing public health issues, Li et al. [61] sought to identify relationships between these two conditions in a population of Chinese police officers. Among these participants, moderate and high occupational stress, as well as high personal strain, were identified as independent risk factors for NAFLD, compared with low occupational stress and low personal strain, which appeared to play a protective role. Of interest, the presence of NAFLD was significantly higher in traffic police officers compared with other members of the police force, drawing attention to the fact that these traffic police officers encounter occupational exposure to traffic noise and air pollution on a full-time basis on most days. As such, this study further suggested that environmental factors may also be implicated in the relationship between NAFLD and occupational stress [61].

Although research in this field remains limited, the aforementioned studies, as well as evidence from animal studies [62], support the existence of direct links between chronic psychological stress and NAFLD. As such, while further research is clearly needed, this evidence suggests that NAFLD may represent a stress-sensitive disorder for which stress management interventions could be beneficial.

## 5. Health Related Quality of Life (HRQoL)

Both physical and mental health problems may have a significant impact on HRQoL. Thus, the assessment of self-reported quality of life can be of significant clinical value in the management of chronic disorders, in order to address both the psychosocial and physical needs of patients [63].

### 5.1. HRQoL and Metabolic Syndrome

Emerging data from a growing number of studies have indicated decreased HRQoL in patients with metabolic syndrome [64,65,66]. For example, when assessing metabolic syndrome as a determinant of HRQoL, a cross-sectional study conducted in Greece identified impaired HRQoL on almost all subscales of the validated Greek version of the 36-item Short Form Survey questionnaire (SF-36) for patients with metabolic syndrome compared with controls [66]. Likewise, an earlier U.S. study identified reduced HRQoL in individuals with metabolic syndrome compared with those without [64]. In contrast, research from Korea suggests a lack of an independent relation to impaired HRQoL in people with metabolic syndrome after adjusting for various confounding variables (e.g., multiple comorbidities), suggesting that the presence of comorbidities may account for the decreased HRQoL among metabolic syndrome patients [67]. However, the findings from a recent systematic review of 30 relevant studies involving 62,063 adults with metabolic syndrome provide further evidence of the positive association between metabolic syndrome and impaired HRQoL [65].

### 5.2. HRQoL and NAFLD

Although data are relatively limited with regard to NAFLD and HRQoL, a number of studies have investigated such a potential relationship. The 29-item Chronic Liver Disease Questionnaire (CLDQ) was utilized in many of these studies, as a disease-specific tool designed to measure HRQoL in individuals with chronic liver disease [68,69]. Indeed, Dan et al. [70] compared HRQoL, as assessed by the CLDQ, in patients with NAFLD and those with chronic hepatitis B or chronic hepatitis C, revealing significantly worse HRQoL in the former for both the overall CLDQ score and most of its subscales [70]. Notably, although a large proportion of NAFLD patients in this study also presented with hypertension, diabetes, and metabolic syndrome, the impact on HRQoL did not appear to be completely explained by these comorbidities [70].

The CLDQ was also utilized in a prospective United States-based study by Tapper and Lai [71] involving 151 adults with a histologic diagnosis of NAFLD [71]. This study, which was designed to investigate whether HRQoL could be improved by a lifestyle-modification intervention (exercise programme along with dietary recommendations for weight loss), revealed that patients with a 5% weight reduction at the 6-month follow-up showed significantly improved HRQoL [71]. Likewise, a recent study by Castellanos-Fernández et al. [72], which incorporated the CLDQ to assess HRQoL among Cuban patients with NAFLD, autoimmune liver diseases, and hepatitis B, documented significantly lower HRQoL for patients with NAFLD and autoimmune liver diseases compared with those with hepatitis B. Interestingly, sleep apnea and abdominal pain were more frequently identified in patients with NAFLD, which may have contributed to reductions in HRQoL among this patient group [72].

Utilizing data from the National Health and Nutrition Examination Survey (NHANES), Golabi et al. [73] assessed HRQoL in 3333 patients with NAFLD compared with 5982 healthy controls. This study invited participants to rate four components of HRQoL over the previous 30-day period and included questions in relation to perceived health status (overall HRQoL), the number of days experienced with physical illness and injury, the number of days within which mental health issues were encountered, and the extent to which physical or mental health had prevented the participant from engaging in usual activities [73]. Notably, participants in the study control group rated their health status as significantly higher than those in the NAFLD group, and were more likely to report an absence of physical health problems. Furthermore, the control group reported significantly fewer occasions whereby physical or mental health issues had prevented engagement in usual activities, indicating that the poorer health status of NAFLD patients may affect their ability to perform normal daily activities. However, it was also suggested that fatigue could have had an impact on the poorer reports of physical health in patients with NAFLD, as this symptom was frequently present among NAFLD patients [73].

Another recent study, which explored the relationship between NAFLD and dietary habits, stress, and HRQoL, reported reduced HRQoL in Korean adults with NAFLD [58]. In this study, HRQoL was assessed by the EuroQol-5D (EQ-5D) instrument, which measures/comprises of five dimensions, namely, mobility, self-care, usual activities, pain/discomfort, and anxiety/depression. This study showed that a decrease in the EQ-5D score by one unit was associated with increased risk of NAFLD by more than threefold, raising the awareness of considering HRQoL in the context of NAFLD [58].

Overall, systematic review data from 14 relevant studies collectively suggest that NAFLD patients have poorer HRQoL in comparison with healthy controls [63]. It is clear that further work is needed in order to address the direct impact of NAFLD on HRQoL, and to gain a further understanding of the underlying mediating factors. Investigating potential predictors of HRQoL in NAFLD patients (e.g., obesity and obstructive sleep apnea), and the extent to which other related comorbidities (e.g., fatigue, depression, and anxiety) may impact the HRQoL could be of clinical importance.

## 6. Potential Underlying Mechanisms Linking NAFLD with Depression, Anxiety, and Stress

A growing burden of disease due to mental health disorders and increasing trends of somatic-psychiatric comorbidity have been noted globally, while patients presenting with multiple diseases are frequently the rule rather than the exception [74,75]. Coexistence of two or more health conditions may be explained by a number of factors, including coincidence without underlying causal connection(s) [74,75,76]. Indeed, surveillance bias may partly explain the fact that patients already receiving care for one chronic disease are more likely to be diagnosed with one or more additional health conditions [76]. However, comorbidity often occurs as a result of true underlying pathophysiologic mechanisms, which may represent direct causal link(s) and/or common risk factor(s) [74,75,76]. An indirect link may also be present between coexisting health conditions, when these have direct causal connection(s) with another common disease/condition [76].

**Figure 1:**
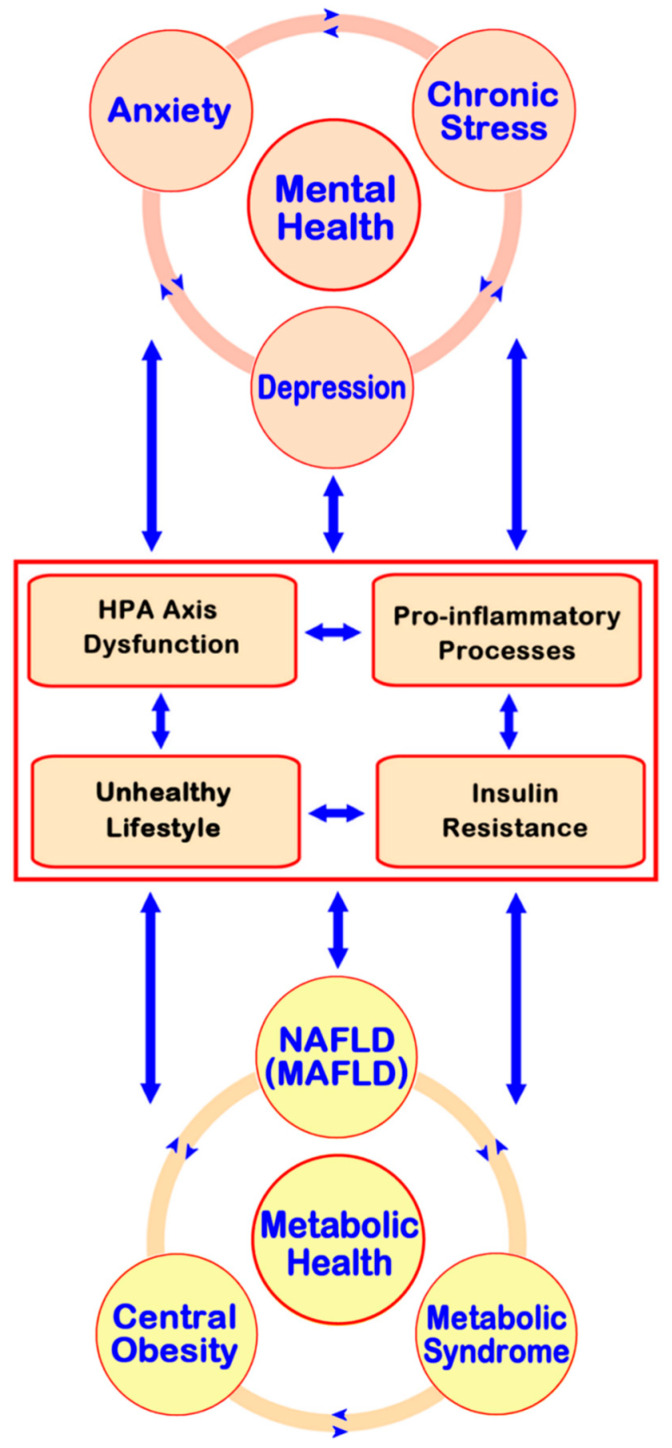
Simplified schematic diagram presenting key potential underlying mechanisms and links between prevalent mental health problems (depression, anxiety and chronic stress) and non-alcoholic fatty liver disease (NAFLD), also referred to as metabolic-associated fatty liver disease (MAFLD). Depending on the duration and the potency of the initial underlying condition, as well as on the individual’s predisposition, one or more vicious cycles may develop over time linking MAFLD and related cardio-metabolic diseases (e.g., central obesity and metabolic syndrome) with mental health comorbidity. Hypothalamic–pituitary–adrenal (HPA) axis dysregulation, pro-inflammatory processes, insulin resistance, and unhealthy lifestyle behaviors (e.g., unhealthy diet and sedentary lifestyle) are among the key mechanisms mediating such potentially bidirectional associations and feed-forward vicious cycles.

As is now well-established for the clustering of cardio-metabolic conditions within the spectrum of the metabolic syndrome [10], the existing evidence indicates that the direct positive associations between mental health disorders (e.g., depression, anxiety, and chronic stress), and both NAFLD and the metabolic syndrome may be attributed to true pathophysiologic links rather than just coincidence [23]. In this context, multiple underlying mechanisms have been proposed to play a role in mediating these associations. The most salient of these potential mechanisms are briefly outlined in this section, while Figure 1 presents these in a simplified schematic diagram.

It is also noteworthy that gender variations in relationship to stress, mental health, and metabolic diseases pose a further issue, since men and women may differ in terms of prevalence and response to these conditions. Indeed, as reported in a study by Choi et al. [44], NAFLD occurs more commonly in men, while depression was almost two times more prevalent in women compared with men. It is further reported that, although disorders such as CVD may be seen as more common in men, certain modifiable risk factors for obesity, metabolic disease, and CVD (e.g., stress) are higher in women [77]. Interestingly, attention has also been drawn to findings that indicate that responses to stress may in fact be similar in men and women, while engaging distinct neural networks [78]. Finally, potential differences between men and women in relevant health behaviors and in the regulation of glucose and energy homeostasis have also been reported [79,80].

### 6.1. Chronic Stress and HPA Axis Dysregulation

Bergmann, Gyntelberg, and Faber [55] suggest that stress can be understood by its division into three components, namely, stressors, the body’s reaction to stressors, and the emotional effects of such reactions. Once the threshold of an individual is exceeded by the sum of applied stressors, the stress response is activated via the HPA axis and the sympathetic nervous system (SNS). Prolongation of this stress response can progressively lead to HPA dysregulation and depression, which could result in subsequent physical illness [32]. Indeed, chronic stress can cause hyper-activation of the HPA axis and the SNS, which, in turn, is associated with obesity (particularly abdominal/central) and metabolic syndrome [23]. Although a number of factors may determine how an individual adapts and responds to stressful situations, the HPA axis represents the major neuroendocrine system responsible for stress regulation via a process regulating the appropriate and timely release of glucocorticoids. This process crucially involves a negative feedback loop, which is of paramount importance in regulating the individual’s response to stress, as it prevents cortisol over-production/secretion. If this inhibitory procedure fails to operate efficiently—for example, under conditions of chronic stress—inappropriate and/or high secretion of cortisol by the adrenals can promote the development of the various metabolic syndrome manifestations, including NAFLD [23]. Of note, cortisol/glucocorticoids increase hepatic gluconeogenesis and blood glucose levels and promote visceral/central fat accumulation, while simultaneously inducing lipolysis (e.g., in subcutaneous adipose tissue depots) and protein degradation (e.g., in skeletal muscle) to provide additional substrates for oxidative pathways in response to stress [23]. These cortisol-induced effects normally stop in a prompt manner upon the removal of the stressor(s), which caused the HPA axis activation. However, chronic stress and over-activation of the HPA axis prolong these metabolically detrimental effects, and can progressively result in increased visceral/central adiposity, insulin resistance, and ectopic accumulation of fat in the liver, all of which are factors contributing to the development and progression of NAFLD [10,23].

### 6.2. Obesity-Related Inflammation and Insulin Resistance

Obesity is associated with, and may lead to, a range of cardio-metabolic, including T2DM, NAFLD and CVD, as well as mental health comorbidities [10]. In this setting, a cyclical relationship with bidirectional links between obesity and psychological health appears plausible, with affective disorders and chronic stress representing risk factors for obesity, and vice versa [10]. Indeed, HPA axis dysfunction resulting from exposure to prolonged stress can result in adipose tissue accumulation leading to obesity, while obesity in itself is a chronic stressful condition that might lead to HPA axis over-activation (e.g., as a consequence of obesity-related chronic inflammatory stress) [22]. This can further lead to increased risk for metabolic syndrome, NAFLD, and CVD [10,22].

To date, a number of studies have identified associations between circulating C-reactive protein (CRP) levels and depression in individuals with obesity [81,82], suggesting that obesity-related pro-inflammatory pathways may play a mediating role in the underlying pathophysiology linking these diseases, including NAFLD. Notably, metabolic syndrome and MDD—which as aforementioned frequently coexist—have been also consistently associated with chronic low-grade inflammation with increased circulating levels of multiple pro-inflammatory factors [13]. There is also evidence for a bidirectional link, whereby an unhealthy lifestyle as a result of depression may increase inflammation, leading to a feed-forward vicious cycle [23,59].

It is further reported that HPA axis dysregulation as a result of negative emotions in response to chronic and/or intense stress could lead to the onset of NAFLD owing to activated hepatic pro-inflammatory processes [9,62]. A study by Russ et al. [9] indicated that inflammation is frequently a prominent feature of NAFLD, which denotes progression to non-alcoholic steatohepatitis (NASH), with a strong identified relationship between NAFLD and psychological stress. Thus, it would appear that pro-inflammatory factors related to psychological distress are also mediators in an important pathway between psychosocial stress and NAFLD [9,13]. Indeed, bidirectional relationships have also been suggested between psychiatric disorders and other chronic immune-mediated inflammatory diseases (e.g., rheumatoid arthritis, multiple sclerosis, ulcerative colitis and Crohn’s disease) [76]. Pro-inflammatory pathways, immune dysregulation, and systemic or multi-organ inflammation are considered to mediate these bidirectional links [76], and these may similarly contribute, at least in part, to the common pathophysiology between certain mental health disorders (e.g., depression, anxiety, and chronic stress) and NAFLD/NASH [10,23].

As well as systemic low-grade inflammation, insulin resistance has been implicated as a potential causal mechanism linking metabolic dysregulation with depressive disorders [83]. As aforementioned, insulin resistance is also included among the detrimental effects of HPA dysfunction, mediated mostly via over-secretion of cortisol by the adrenals and of adipokines/cytokines by the accumulated excess and/or ectopic adipose tissue [10,13,23]. Overall, there appears to be an interaction between insulin resistance, psychological health, and NAFLD [58]. Interestingly, in testing the hypothesis that insulin resistance or inflammation might partially explain the relationship between depression and NAFLD, Lee et al. [84] were unable to identify such a link when utilizing CRP as a biomarker. However, a 40–50% increased risk of NAFLD in individuals with depression was identified in this study, whereby depression appeared to be linked to insulin resistance (rather than to inflammation), suggesting that insulin resistance may be driving the increased risk for NAFLD.

### 6.3. Gut Microbiome Dysbiosis in Metabolic Diseases and Its Association with Psychological Disorders

Bacteria within the gut are now recognised to play a major role in the bi-directional communication between the gastrointestinal tract and the brain, which seems to be mediated via intricate mechanisms and pathways [85,86]. This is supported by a growing body of emerging data indicating that the gut microbiome plays an important role in both physical and mental health through a number of bi-directional processes and appears to be associated with a number of conditions, including neuro-psychiatric, psychological, and physiological disorders [85]. Of note, lifestyle factors affect the maintenance of the normal gut microbiota, with diet being of particular importance, as is evident from both animal and human studies [85]. Indeed, the gut microbiome is influenced by dietary factors, such as low fibre intake, which can result in dysbiosis and inflammatory processes that may impact both mental and physical health [85]. In this context, the dysbiosis of the gut microbiota is shown to have a number of effects on metabolism, which can affect the body weight [87]. For example, Westernised dietary habits are implicated in the pathogenesis of obesity and may lead to an imbalance in gut microbiota, which can further contribute to obesity-related complications via a number of processes, including endocrine, neurochemical, and inflammatory alterations [88]. As such, it appears that gut microbiota may also be implicated in the aetiology of NAFLD, particularly as certain microbiota enterotypes appear to exhibit a greater ability to absorb energy from the diet and seem to be linked to higher total body fat [89]. Finally, there are also established relationships between obesity and mental health that may be connected to changes in gut microbiota, resulting in such a comorbidity [88]. Accordingly, the gut microbiome dysbiosis in obesity-related cardio-metabolic diseases may be further implicated in the pathophysiology between NAFLD and mental health disorders, such as depression, anxiety and chronic stress, and dietary changes, which can address this dysbiosis may potentially lead to improvements in both physical and psychological health.

## 7. Future Research Directions and Concluding Remarks

NAFLD has strong associations with metabolic syndrome and represents a growing and important public health issue globally. The studies outlined in this review, and summarized in Table 1 and Table 2, link NAFLD—directly or indirectly (via a strong overlap with the metabolic syndrome)—with prevalent mental health problems, such as depression, anxiety, and chronic stress, thus expanding the spectrum of its potential pathophysiologic associations. Indeed, the relevant existing evidence indicates that further aspects relating to mental health and HRQoL may have clinical implications for the management of patients with NAFLD, and hence are worthy of more thorough investigations. However, there are certain difficulties associated with such work owing both to the complex nature of affective disorders, and to the substantial overlap of NAFLD with multiple other conditions related to obesity and the metabolic syndrome. For example, there are different types of depression (e.g., MDD and bipolar) and anxiety (e.g., general, social, panic disorder, and phobias) that increase the complexity of the relevant diagnoses and management [90,91,92,93]. In addition, there are a number of validated tools for assessing depression and anxiety in primary care settings, with the PHQ-9 and GAD-7 proving particularly popular [94,95,96], but practical issues (e.g., time constraints) could also contribute to the under-detection of such conditions. Likewise, stress can be broken down into a range of different types representing a complex impact (e.g., physical or psychosocial stress, as well as post-traumatic, occupational/workplace, or financial stress) [90,97]. Interestingly, studies involving animal models have provided evidence that changes in behavior may differ depending on the type of stress experienced [98]. Again, there are various tools for measuring stress, with the Perceived Stress Scale being particularly widely used [99]. Therefore, it should also be highlighted that, although almost all of the studies presented in this review utilized validated instruments for assessing mental health comorbidity (depression, anxiety and stress), these self-reported measures may not fully capture the details required for precise diagnosis and/or severity assessment of the corresponding underlying condition(s). Hence, it is suggested that relevant future studies should also incorporate structured diagnostic methods for the precise assessment of mental health comorbidity, in addition to participant-reported measures [40,84].

Additionally, it is important to consider the issue of multi-morbidity and overlapping symptoms. Certain studies indicate a potential independent association between mental health and NAFLD [46,70], while others report on the presence of sleep disorders and fatigue in NAFLD patients, which might have an impact on responses to self-report measures related to mental health [72,73]. Furthermore, most of the existing studies in this field utilized cross-sectional research designs, which, although useful for identifying associations, are unable to provide information regarding causality. As such, given the relationship with other comorbidities and the difficulties in determining causality, future studies employing a longitudinal design may prove fruitful in attempting to overcome this issue [44].

Of further note, as weight gain is implicated in the progression of insulin resistance and NAFLD, consideration should be given to the potential weight gain side effects of certain medications for mental health disorders (e.g., certain antidepressants) [10,89]. Indeed, a review by Hasnain and Vieweg [100] highlights the weight changing effects of certain medications used to treat a number of mental health conditions (e.g., mood stabilizers, antidepressants, and anti-anxiety medications), and draws attention to the potential benefits of switching such medications in situations whereby weight gain becomes troublesome, taking into consideration both the patient’s condition and possible side effects. Notably, a recent systematic review suggests that metabolic disorders are often exacerbated in people taking antidepressant or antipsychotic medications, with most of the reviewed studies showing a 5% weight gain in individuals under antidepressant therapy [101]. Considering such data, and given the frequency with which antidepressants are currently prescribed, it becomes evident that, although the weight gain risk may be slightly lower with antidepressants compared with antipsychotic drugs [102,103], it is important to also consider the potential weight gain adverse effects of drugs prescribed for mental health conditions, particularly when investigating associations between mental health and obesity-related cardio-metabolic disorders, such as NAFLD.

Based on the broader relevant literature, additional parameters, such as emotional loneliness, may also be involved in the noted associations between mental health problems and NAFLD [32]. Thus, a broader understanding of the individual’s circumstances and coping strategies may prove valuable [104,105]. Indeed, it is important to consider a broad number of biological and psychological underlying factors that might play a role in the interplay of associations between affective disorders and NAFLD. For example, in future research, the notion of social support and coping strategies should be taken into consideration, given their associations with physical and psychosocial outcomes for a range of disorders. Interestingly, a recent study by Funuyet-Salas et al. [106]—which attempted to identify factors that might have an influence on HRQoL, mental health status, and coping strategies in a population of Spanish individuals with NAFLD—showed a higher risk of psychosocial problems in female patients who indicated low levels of perceived social support and who presented with significant fibrosis. As such, the study authors suggested that factors relating to mental health, HRQoL, and coping strategies could play a role in the management of patients with NAFLD, and that the potential advantages of relevant psychological interventions should be considered in clinical practice [106].

Overall, despite its increasing prevalence globally, NAFLD has received much less attention than other chronic non-communicable diseases with respect to potential bidirectional links to mental health and HRQoL. Of note, for obesity-related diseases, such as NAFLD, there is potentially an additional element of a self-blame perception, which may also lead to shame and stigma, preventing the individual from seeking appropriate help [1]. As such, there is a current discussion regarding the potential benefits of changing the relevant terminology from NAFLD to MAFLD (metabolic-associated fatty liver disease), following a recent consensus of international experts [107,108]. In this context, it is argued that the current NALFD terminology carries certain negative messages and could be associated with stigmatization and trivialization. This may be addressed by adopting the term MAFLD, which could potentially lead to a reduction in the existing negativity, and might aid in better understanding/re-defining this highly prevalent disorder.

In conclusion, NAFLD is a growing problem globally, which deserves further attention for certain aspects of its pathophysiology, particularly regarding underlying links to depression, anxiety, and stress. Elucidating such associations may help the prevention and/or management of NAFLD in routine clinical practice, especially as specific NAFLD treatments are currently lacking. Notably, it is possible that a feed-forward vicious cycle exists between these prevalent mental health conditions and NAFLD, whereby depression, anxiety and/or chronic stress may promote NAFLD, and vice versa. Recognizing and breaking this potential vicious cycle in the clinical practice, as well as strengthening mental health awareness in NAFLD-related primary and secondary care pathways, may have substantial benefits for the interventions against NAFLD.

A key limitation of the present review is that the relevant searches focused only on two databases (PubMed and Google Scholar) and on papers published only in English mostly within the past decade, thus relevant papers not meeting these criteria may have been omitted. Nonetheless, the present review highlighted important existing evidence pertaining to linkages between the highly prevalent mental health problems of depression, anxiety, and chronic stress and both NAFLD and metabolic syndrome. Furthermore, as aforementioned, causation is difficult to determine in this context, and there might be various reasons why two or more conditions may co-exist [26]. As there is no health and wellbeing without mental health [109], further research is clearly needed in this field, and could help in the development of appropriate complex interventions for addressing the interrelated underlying pathophysiologic mechanisms and risk factors. Such interventions should also incorporate a theoretical framework to promote the incorporation of a compassionate approach, further targeted towards addressing the patient’s individual lifestyle, confidence levels, and available coping strategies, and aimed at reducing any perceived stigma that such patients might experience.

## Figures and Tables

**Table 1:** Selected studies on links between metabolic syndrome and mental health wellbeing.

Study [Reference]	Country	Study Design/Cohort	Mental Health Related Outcome Assessed and Method	Outcome/Main Findings
Tang, Wang, & Lian (2016) [34]	China	Systematic review and meta-analysis 18 cross-sectional studies N = 41,168 2 cohort studies N = 1388	Anxiety Various	Supports a significant relationship between anxiety and metabolic syndrome.
Akbari et al. (2017) [39]	Iran	Cross-Sectional Community-based cohort N = 470 (male: 50.2%) Mean Age: 55.7 ys	Depression/Anxiety HADS	No significant association between depression, concurrent depression, and anxiety with metabolic syndrome. Patients with anxiety had lower prevalence of metabolic syndrome than healthy subjects.
Butnoriene et al. (2018) [32]	Lithuania	Cross-Sectional Metabolic syndrome: N = 384 (female: 53.6%) Mean Age: 63.7 ys Without Metabolic Syndrome: N = 731 (male: 52.5%) Mean Age: 61.1 ys	Depression/Anxiety HADS MINI	Depression and anxiety was identified as being more common in individuals with metabolic syndrome in comparison with those without.
Dunbar et al. (2008) [35]	Australia	Cross-Sectional N = 409 (male: 50.9%) Mean Age: 60.5 ys	Depression/Anxiety HADS Kessler 10	Results show an association between metabolic syndrome and depression, but not with psychological distress and anxiety.
Shinkov et al. (2018) [33]	Bulgaria	Cross-sectional N = 2111 (female: 54.7%) Mean Age: 46.4 ys	Depression/Anxiety Zung Self-Rating Depression and Anxiety Scales	Depression and anxiety scores were higher for those with metabolic syndrome.
Skilton Moulin et al. (2007) [36]	France	Observational N = 1598 (male: 62.9%) Mean Age: 51.8 ys	Depression/Anxiety HADS	Metabolic syndrome was associated with depressive symptoms, but not with anxiety in men and women, irrespective of body weight.
Kuo et al. (2019) [56]	USA	Systematic review and meta-analysis 30 studies N = 67,037	Stress Various	Different sources of frequently occurring stress should be investigated. Usual methods (e.g., lifestyle change) for preventing metabolic syndrome, may be less effective if psychosocial stress is overlooked.
Ford et al. (2008) [63]	USA	Cross-Sectional Metabolic syndrome: N = 737 (female: 50.4%) Mean Age: 52.2 ys Without metabolic syndrome: N = 1122 (female: 50.5%) Mean Age: 41.8 ys	HRQoL Centers for Disease Control and Prevention HRQOL-4 tool	HRQoL was worse in people with metabolic syndrome compared with those without.
Lee et al. (2012) [66]	Korea	Cross-sectional Metabolic Syndrome: N = 2635 (male: 56%) Mean Age: 52.8 ys Without Metabolic Syndrome: N = 6306 (male: 52.3%) Mean Age: 40.8 ys	HRQoL EQ-5D EQ VAS	Unable to identify and independent association in relation to impaired HRQoL in people with metabolic syndrome after adjusting for confounding variables.
Saboya et al. (2016) [64]	Brazil	Systematic review 30 studies N = 62,063	HRQoL SF-36 was the most frequently utilized	Evidence suggests association between metabolic syndrome and poorer HRQoL.
Tziallas et al. (2012) [65]	Greece	Cross-Sectional Metabolic Syndrome: N = 206 (female: 54.8%) Mean Age: 58.4 ys Controls: N = 153 (male: 53%) Mean Age: 50.1 ys	HRQoL SF-36	Study identified impaired HRQoL on almost all subscales of the SF-36 for patients with metabolic syndrome compared to controls.

EQ-5D: EuroQoL 5 dimensions; EQ VAS: EuroQol visual analogue scale; HADS: Hospital Anxiety and Depression Scale; HRQOL: Health-Related Quality of Life; MINI: Mini International Neuropsychiatric Interview; PHQ-9: Patient Health Questionnaire; SF-36: Short Form-36; ys: years.

**Table 2:** Selected studies on links between non-alcoholic fatty liver disease (NAFLD) and mental health comorbidity.

Study [Reference]	Country	Study Design/Cohort	Mental Health Related Outcome Assessed & Method	Outcome/Main Findings
Bashir, Shafi, & Khalil (2020) [44]	Pakistan	Observational Newly US-diagnosed NAFLD N = 170 (male: 64.1%) Mean Age: 39.8 ys	Depression PHQ-9	24.1% of NAFLD patients suffered from clinical depression, implying frequency of depression in people with NAFLD.
Cho et al. (2021) [46]	Korea	Longitudinal N = 142,005 (female: 64.6%) Mean Age: 35.6 ys	Depression CES-D	Depression was associated with increased risk of incident hepatic steatosis and higher probability of advanced liver fibrosis, particularly among individuals with obesity.
Jung et al. (2019) [42]	Korea	Observational N = 112,797 (female: 51.5%) Mean Age: 40 ys Degree of NAFLD assessed by US, FLI, and FIB-4	Depression CES-D	Evidence of an association between the severity of NAFLD and depression.
Kim et al. (2019) [45]	USA	Cross-sectional N = 10,484 (female: 51.2%) Mean Age: 47 ys NAFLD defined by USFLI, HSI, and FLI	Depression PHQ-9	Study identified a higher prevalence of depression among individuals with NAFLD compared to those without.
Sayiner et al. (2020) [48]	USA	N = 30,908,679 (random 5% sample of Medicare data from 2005–2016; female: 54.59%) Mean Age: 70.11	Depression Based on ICD-9 and ICD-10 codes	Depression identified as one of the most common extrahepatic diseases associated with NAFLD.
Tomeno et al. (2015) [24]		Intervention study N = 258 NAFLD comorbid with MDD: N = 32 (male: 56.2%) Mean Age: 46.5 ys NAFLD without MDD: N = 226 (male: 52.6%) Mean Age: 50.7 ys NAFLD diagnosis based on liver biopsy	Depression MDD diagnosed according to the DSM-IV criteria.	Following a 48-week lifestyle intervention, patients with NAFLD demonstrated a poor response with less effective treatment outcomes.
Weinstein et al. (2011) [40]	USA	Retrospective NAFLD: N = 184 (female: 69.4%) Mean Age: 46.7 ys Hepatitis B: N = 190 (male: 61.9%) Mean Age: 43.6 ys Hepatitis C: N = 504 (male: 59.7%) Mean Age: 48.6 ys	Depression Self-reported Diagnosis confirmed by history of prescription medication.	Study identified a higher prevalence of depression in patients with NAFLD and HCV, compared with patients with HBV and members of the general population.
Xiao et al. (2021) [49]	Singapore	Systematic review and meta-analysis including 10 studies N = 2,041,752	Depression Various	High prevalence of depression in NAFLD patients was identified. Risk factors include BMI, diabetes, and being female.
Choi et al. (2021) [43]	South Korea	Retrospective Cross-Sectional N = 25,333 (male: 56.2%) Mean Age: 47 ys US-diagnosed NAFLD prevalence: 30.9%	Depression/Anxiety BDI State-Trait Anxiety Inventory	Severe steatosis is significantly related to both state and trait anxiety in patients with NAFLD. When gender was taken into consideration, a 44% increase in the risk of depression among women with NAFLD was demonstrated.
Labenz et al. (2020) [47]	Germany	Retrospective cohort study Patients with NAFLD: N = 19,871 (male: 57.5%) Mean Age: 58.5 ys Patients without NAFLD: N = 19,871 (male: 57.5%) Mean Age: 58.5	Depression/Anxiety ICD-10 Codes	Study highlighted a significant association between NAFLD and development of depression/anxiety was noted, independent of comorbidities.
Youssef et al. (2013) [41]	USA	Cross-sectional N = 567 (female: 67%) Mean Age: 48 ys NAFLD diagnosis based on liver biopsy	Depression/Anxiety HADS	Symptoms of depression and anxiety common in patients with NAFLD. Study identified a positive association between greater hepatocyte ballooning and depression in patients with NAFLD.
Han (2020) [57]	Korea	Cross-Sectional Total: N = 17,726 (male: 50.6%) Mean Age: 43.9 ys HSI ≥ 36: N = 3764 (male: 61.3%) Mean Age: 45.1 ys HSI < 36: N = 13,962 (female: 52.5%) Mean Age: 43.5 ys NAFLD definition based on an HSI value ≥ 36	Stress EQ-5D Stress perception rate	Decrease in the EQ-5D score by one unit increased the risk of NAFLD by more than threefold. Indication of an increased risk of NAFLD (by 1.3 times) in individuals with increased perceived stress.
Kang et al. (2020) [55]	Korea	Cross-sectional Total: N = 171,321 (male: 50.1%) Mean Age: 39.8 ys With NAFLD: N = 47,538 (male: 76.6%) Mean Age: 42 ys Without NAFLD: N = 123,783 (female: 60.1%) Mean Age: 38.9 ys NAFLD diagnosed by ultrasonography	Stress PSI	An independent association between higher levels of perceived stress and a greater prevalence of NAFLD was identified.
Li et al. (2016) [60]	China	Cross-sectional Total: N = 2367 (male: 100%) Mean Age: 36.65 ys NAFLD new onset: N = 739 (male: 100%) Mean Age: 36.9 ys NAFLD non-onset: N = 1628 (male: 100%) Mean Age: 36.54 ys	Stress OSI-R	High occupational stress and high personal strain identified as independent risk factors for NAFLD. Presence of NAFLD was significantly higher in traffic police officers.
Russ et al. (2015) [8]	UK	Meta-analysis of Individual Study Participants N = 166,631 (female: 55%) Mean Age: 46.6 ys	Stress GHQ	Psychological distress associated with liver disease mortality.
Assimakopoulos et al. (2018) [62]	Greece	Systematic review 14 studies N = 5000	HRQoL Various	NAFLD patients have poorer HRQoL compared with healthy controls.
Castellanos-Fernández et al. (2020) [71]	Cuba	Cross-sectional NAFLD: N = 221 (female: 67.9%) Mean Age: 54 ys Hepatitis B: N = 91 (male: 56%) Mean Age: 45.9 ys AILD: N = 43 (female: 90.7%) Mean Age: 49.3 ys	HRQoL CLDQ	CLDQ and HRQoL scores were significantly lower for patients with NAFLD and AILD compared with HBV.
Dan et al. (2007) [69]	USA	Observational NAFLD: N = 106 (female: 69.8%) Mean Age: 46.4 ys Hepatitis B: N = 56 (male: 73,2%) Mean Age: 45.4 ys Hepatitis C: N = 75 (male: 57.3%) Mean Age: 47.0 ys	HRQoL CLDQ	Patients were identified from the Liver Disease Quality of Life Database. HRQoL scores were significantly lower for NAFLD patients compared with patients with hepatitis B or hepatitis C on multiple CLDQ domains.
Golabi et al. (2016) [72]	USA	Cross-sectional NAFLD: N = 3333 (male: 54.5%) Mean Age: 51.31 ys Controls: N = 5982 (male: 51.8%) Mean Age: 47.5 ys NAFLD was determined by the FLI	HRQoL HRQOL-4	NAFLD is associated with impaired HRQoL.
Tapper & Lai (2016) [70]	USA	Prospective longitudinal study N = 151 (male: 60%) Mean Age: 51.5 ys NAFLD diagnosis: histologic	HRQoL CLDQ	At the 6-month follow-up of a weight loss intervention programme, this study revealed that patients achieving a 5% weight reduction showed significantly improved HRQoL.
Funuyet-Salas et al. (2020) [92]	Spain	Cross-sectional N = 492 (male: 58.9%) Mean Age: 54.9 ys Biopsy-proven NAFLD	Multiple SF-12 CLDQ-NAFLD HADS BD-II COPE-28 MSPSS	Low perceived social support, significant fibrosis, and female sex were independently associated with a higher-risk psychosocial profile in NAFLD.

AILD: Autoimmune liver disease; BDI: Beck Depression Inventory; BD-II: Beck Depression Inventory II; CES-D: Centre for Epidemiological Studies—Depression; CLDQ: Chronic Liver Disease Questionnaire; COPE-28: Brief COPE (28-item self-reporting measure of coping styles in response to a stressful experience); DSM-IV: Diagnostic and Statistical Manual of Mental Health Disorders IV; EQ-5D: EuroQol 5 dimensions; FIB-4: fibrosis-4 score; FLI: fatty liver index; GHQ: general health questionnaire; HADS: Hospital Anxiety and Depression Scale; HSI: hepatic steatosis index; HRQOL: Health-Related Quality of Life; HBV: hepatitis B virus; HCV: hepatitis C virus; ICD: International Classification of Diseases; MSPSS: Multidimensional Scale of Perceived Social Support; OSI-R: Occupational Stress Inventory-Revised; PHQ-9: Patient Health Questionnaire; PSI: perceived stress inventory; SF-12: 12 item Short Form Health Survey; US: ultrasonography; USFLI: US fatty liver index; ys: years.

## Data Availability

Not applicable.
